# *Aedes* (*Stegomyia*) *albopictus*’ dynamics influenced by spatiotemporal characteristics in a Brazilian dengue-endemic risk city

**DOI:** 10.1016/j.actatropica.2016.10.010

**Published:** 2016-12

**Authors:** Juliana M.T. Bezerra, Raphaela G.P. Araújo, Fabrício F. Melo, Caroline M. Gonçalves, Bárbara A. Chaves, Breno M. Silva, Luciana D. Silva, Silvana T. Brandão, Nágila F.C. Secundino, Douglas E. Norris, Paulo F.P. Pimenta

**Affiliations:** a Medical Entomology Laboratory, René Rachou Research Centre – FIOCRUZ-MG, Avenue Augusto de Lima, 1715, Barro Preto, Belo Horizonte, Minas Gerais, 30190-002, Brazil; b Tropical Medicine Foundation Dr. Heitor Vieira Dourado, Manaus, Avenue Pedro Teixeira, 25, Dom Pedro, Manaus – AM, Amazonas, 69040-000, Brazil; c Department of Biological Sciences, Federal University of Ouro Preto, Campus Morro do Cruzeiro Bauxita, Ouro PretoOuro Preto, Minas Gerais, 35400-000, Brazil; d Department of Internal Medicine, Faculty of Medicine, Federal University of Minas Gerais, Avenue Alfredo Balena, 190, Belo Horizonte, 30130-100, Brazil; e Belo Horizonte Secretary of Health, Avenue Afonso Pena, 2336, Funcionários, Belo Horizonte, Minas Gerais, 30130-007, Brazil,; f Department of Molecular Microbiology and Immunology, Johns Hopkins Malaria Research Institute, Johns Hopkins University Bloomberg School of Public Health, Wolfe Street, Baltimore, 615 N, Maryland, MD 21205, USA

**Keywords:** *Dengue virus* (DENV), Field-caught *Aedes albopictus*, Epidemic, Population distribution, Infection rate

## Abstract

Brazil reported the majority of the dengue cases in Americas during the last two decades, where the occurrence of human dengue cases is exclusively attributed to the *Aedes* (*Stegomyia*) *aegypti* (Linnaeus). Nowadays, other recognized *Dengue virus* (DENV) vector in Asian countries, *Aedes* (*Stegomyia*) *albopictus* (Skuse), has been detected in more than half of the 5565 Brazilian municipalities. Therefore, the aim of the present study was to investigate the presence of, and determine the *Ae. albopictus*’ dynamics influenced by spatiotemporal characteristics in a dengue-endemic risk city of Belo Horizonte, Minas Gerais State’s capital. *Aedes albopictus* were collected across four consecutive DENV transmission seasons from 2010 to 2014. These mosquitoes were caught in three selected districts, which had been reported in the previous ten years as having high mosquito densities and an elevated concentration of human dengue cases during epidemic seasons. All field-caught *Ae. albopictus* was individually processed by real-time RT-PCR, to research the DENV presence. The third season (p < 0.05) and the Pampulha district (p < 0.05) had the highest proportions of field-caught *Ae. albopictus*, respectively. The second season had the highest proportion of DENV-infected field-caught females (p < 0.05), but there was no difference among the proportions of DENV-infected *Ae. albopictus* when comparing the collection in the three districts (p = 0.98). Minimum (p = 0.004) and maximum (p < 0.0001) temperature were correlated with the field-caught *Ae. albopictus* in four different periods and districts. In the generalized linear model of Poisson, the field-caught DENV-infected *Ae. albopictus* (p = 0.005), East district (p = 0.003), minimum temperature (p < 0.0001) and relative humidity (p = 0.001) remained associated with the total number of human dengue cases. Our study demonstrated that the number of field-caught DENV-infected *Ae. albopictus* was inversed correlated with the number of human dengue cases. Our study raises the possibility that the DENV circulating in mosquitoes *Ae. albopictus* is happening in non-epidemic periods, showing that this species may be keeping only the presence of the virus in nature. Further long-term studies are necessary to better understand the role of *Ae. albopictus* in DENV transmission and or its vectorial competence in Belo Horizonte and in other endemic cities in Brazil and in the New World countries.

## 1 Introduction

Dengue is a tropical disease and the most important mosquito-vectored viral infection of humans being, affecting many tropical and sub-tropical countries in Southeast Asia, the Pacific and the Americas. Approximately 50 million people are infected with dengue each year and, about 500,000 of them are hospitalized with dengue hemorrhagic fever, the severe form of the disease. In recent years, the annual average of dengue cases reported to the World Health Organization (WHO) has increased dramatically. In 2013, countries in the Americas reported more than 2.3 million cases of dengue, with 37,692 cases of severe dengue and 1280 deaths, resulting in a mortality rate of about 0.05% (PAHO, 2014, WHO, 2014). Brazil reported the majority of the dengue cases during the last two decades with 1,452,489 cases reported in 2013 (MS, 2015). Nowadays, dengue virus (DENV) transmission has been reported in all 27 Brazilian states (MS, 2015). The focus of this study was the endemic city of Belo Horizonte, capital of Minas Gerais State, located in southeast Brazil. Belo Horizonte has approximately 2.3 million inhabitants. In 1996, the first dengue cases were confirmed in the southeastern city of Belo Horizonte. In the following years, the city has recorded thousands of autochthonous cases of dengue every year transforming the city in a dengue-endemic risk area. The recent and worst dengue epidemic in the city history was 2013 with 89,213 cases reported (, 2015).

Understanding the relationship between DENV and mosquito vectors is critical for epidemiological reasons. The primary dengue vector in the Americas is the *Aedes aegypti* mosquito. However, in recent decades *Ae. albopictus*, the “Asian Tiger mosquito” native to Southeast Asia, has invaded most of the Americas, from the United States to Argentina (Lambrechts et al., 2010). At present, the colonization of *Ae. albopictus* has been confirmed in 34 countries (Kraemer et al., 2015). *Aedes albopictus* is the main vector of DENV in rural and semi-urban tropical Asia and has caused small epidemics in Europe. This mosquito can also transmit the *Flavivirus* and the *Alphavirus* agents of yellow fever, West Nile and Chikungunya (Hawley, 1988, Delatte et al., 2008, Paupy et al., 2009, Tomasello and Schlagenhauf, 2013). Laboratory studies have shown that *Ae. albopictus* could be infected with more than 20 types of arboviruses, drawing attention to its broad vector competence, although its role in natural transmission is uncertain (Gubler et al., 2001, Vega-Rua et al., 2013). Most relevant for our work, Pessanha et al. (2011) and Martins et al. (2012) demonstrated the occurrence of DENV-infected *Ae. albopictus* larva in Brazilian cities including in the city of our study. Moreover, studies have shown that Brazilian populations of *Ae. albopictus* from both urban and rural areas, are susceptible to DENV infection in the laboratory (Castro et al., 2004, Fernandes et al., 2004).

Government health authority-driven vector control programs are the only available tools for the control of dengue disease in much of the endemic world. There are currently no drugs or vaccines available to cure or prevent DENV infection in humans. The presence and the density of the primary vector *Ae. aegypti* usually determines the necessity for vector control actions during dengue epidemics. Oral infection experiments with the four serotypes have also demonstrated that Asian *Ae. albopictus* are more susceptible to DENV infection that the “traditional” vector *Ae. aegypti* (Rudnick and Chan, 1965, Richards et al., 2012, Vega-Rua et al., 2013). Even though *Ae. albopictus* has successfully invaded much of the Americas, its role in DENV transmission is not well defined, despite laboratory studies that have found that *Ae. albopictus* can be successfully infected by all DENV serotypes (Guo et al., 2013, Mutsuo et al., 2014). In contrast to *Ae. aegypti*, *Ae. albopictus* is not considered a target by governmental programs for dengue vector control in American countries.

A recent study provided the first geographical distribution of *Ae. albopictus* in Brazil at the municipal level with the mosquito being detected in more than half of all Brazilian municipalities (59.0%) (Carvalho et al., 2014). The highest reported HI (house index) values are in municipalities and states located in the southeast (Carvalho et al., 2014). Its presence and broad distribution in Brazilian dengue endemic cities has never been correlated with epidemics. Even so, the discovery of DENV-infected larvae of *Ae. albopictus* in the Brazilian states of Minas Gerais, Pernambuco and Roraima, located respectively in the southeast, northeast and west of the country, has drawn attention (Pessanha et al., 2011, Antunes et al., 2014, Deininger et al., 2014, Villela and Natal, 2014). In 2012, Martins et al. (2012) found for the first time *Ae. albopictus* adults infected with DENV in Fortaleza, the capital city of the northeast State of Ceará, which has raised the level of concern even further.

The goal of this study was to verify the presence of, and determine the *Ae. albopictus*’ dynamics influences by spatiotemporal characteristics in dengue-endemic risk city of Belo Horizonte, capital of the State of Minas Gerais. Data for *Ae. albopictus* were collected across four consecutive DENV transmission seasons from 2010 to 2014 independently of *Ae. aegypti*, the primary vector in the New World. The data presented here illustrates that *Ae. albopictus* should be taken into account in future studies aimed at understanding dengue transmission dynamics and in developing effective strategies for dengue vector control in large urban settings.

## 2 Methods

### 2.1 Study area

This research was conducted in the Brazilian city of Belo Horizonte, capital of Minas Gerais State, located at latitude 19°49′01″, longitude 43°57′21″ and altitude 858 m above sea level. The city has an area of 331,400 km^2^, with a population of 2,375,151 inhabitants and population density of 7,167.02 inhab./km^2^ (IBGE, 2014). The climate is tropical to subtropical with a marked dry season, with temperatures ranging from 18.0 °C to 23.0 °C and an annual average of 21.1 °C. Winter is characterized by low temperatures and little precipitation and summer by high temperatures and rainfall (PBH, 2015). The DENV transmission season occurs in the summer and is typified by high relative humidity near to 65.0% with most of the average annual rainfall of approximately 1500 mm falling between October and March (INMET, 2016). *Aedes albopictus* were caught in three selected districts, which had been reported in the previous ten years as having high mosquito densities by the City Health Department and a high concentration of dengue cases during epidemic seasons (PBH, 2015). Sixty collection locations were randomly selected using the MapInfo Professional 2009 version 10.0 (MapInfo^®^, USA) program based on the city map of selected districts.

### 2.2 Mosquito collections

Adult female *Ae. albopictus* were caught along with other mosquito species including *Ae. aegypti* during the DENV transmission seasons (October from one year to May of the following year) in the four-year study period. There were no collections in the dry-winter seasons (June to September) when the presence of mosquitoes is not detectable in the city. BG-Sentinel Full Version^®^ traps (Biogents AG, Germany) were used to collect adult host-seeking mosquitoes. The BG-Sentinel trap is considered a gold standard instrument for the detection, capture and surveillance of the *Ae. albopictus* mosquito (Williams et al., 2007; Farajollahi et al., 2009, Werner et al., 2012, Crepeau et al., 2013) including in Asian countries where this species is the natural main vector of dengue (Gratz, 2004). A single trap was placed at twenty *peri*-domestic locations in each of the three selected districts, making a total of 60 trap locations for the study. The 60 traps were placed on Mondays and the mosquitoes were collected in 24 h time-intervals on Tuesdays, Wednesdays and Thursdays of each week across the four-year period of this study. On each collection day, mosquitoes were individually separated into tubes, the collection date, species, district, and detailed trap location recorded, and specimens stored at −70 °C for subsequent molecular analyses. All individual field-caught mosquitoes classified as *Ae. albopictus* were checked for their GPS-district location, quantified, visually checked for absence of blood meal under stereoscope and analyzed for DENV infection (Gonçalves et al., 2014).

### 2.3 DENV detection by real-time RT-PCR

All field-caught *Ae. albopictus* were carefully re-checked under a stereoscope to confirm the species identification and the absence of blood meal. RNA extraction and DENV detection by real-time RT-PCR were conducted as previously reported (Gonçalves et al., 2014). In brief, whole bodies of field-caught *Ae. albopictus* were macerated individually in 1.5 ml conical tubes in 200 μl PBS. After centrifugation, 140 μl of the supernatant was processed for RNA extraction using the QIAamp^®^ Viral RNA Mini Kit (Qiagen^®^, Venlo, Limburgo) according to the manufacturer's instructions. DENV detection was performed by real-time RT-PCR in an ABI Prism 7500 Fast Real Time PCR machine using the Power SYBR^®^ Green RNA-to-C_T_™ 1-Step system (Applied Biosystems, California, USA). The 3’non-coding region primers, B1-forward (5′-AGGACYAGAGGTTAGAGGAGA-3′) and B2-reverse (5′-CGYTCTGTGCCTGGAWTGAT-3′), used in this study were designed based on the study of Leparc-Goffart et al. (2009) with minor modifications. All analyses were performed in duplicate and in parallel with standardized samples (to provide standard curves), positive controls and negative controls. Results indicative of DENV presence in each mosquito were obtained as previously described (Gonçalves et al., 2014) using a standard curve and analyzing the melting curve for specificity of the amplified products (melting point ∼78.6 °C) and CT of 35, according to rigorous criteria from literature for The Minimum Information for Publication of Quantitative Real-Time PCR Experiments (MIQE) (Bennett et al., 2002).

### 2.4 Infection rate and evaluation of relative abundance

The infection rate (IR) was calculated as the proportion (percentage) of *Ae. albopictus* in which DENV was detected by real-time RT-PCR and it was related to the total quantity of *Ae. albopictus* mosquitoes caught in each DENV transmission season and/or each district. The relative abundance (RA) of *Ae. albopictus* was calculated by comparing the number of field-caught *Ae. albopictus* in distinct districts. In contrast, the RA of DENV-infected *Ae. albopictus* was calculated by comparing the quantity of infected and non-infected mosquitoes of this species.

### 2.5 Collection of dengue human cases

The correlation of the number of human cases with infected field-caught *Ae. albopictus* was possible due to the information about confirmed dengue cases collected in the Belo Horizonte Secretary of Health homepage (PBH, 2014). The confirmed human cases are patients that were clinical diagnosed with dengue but also had been validated by laboratory diagnosis that is based on virus isolation and serology following strict rules determined by the Brazilian Ministry of Health (MS, 2015).

### 2.6 Climatic factors

Meteorological data for rainfall,temperature (minimum, maximum and average) and relative humidity of Belo Horizonte, were obtained by the daily consultation on the website of the National Institute of Meteorology (INMET), in the Automatic Stations Module. Municipal information was fed into the homepage through the Pampulha Meteorological Station. The information was accessed by the Internet address [http://www.inmet.gov.br/sonabra/pg_dspDadosCodigo.php?QTUyMQ==] throughout the study period.

### 2.7 Statistical analysis

Data were entered into an access database, verified by double entry and analyzed using the Statistical Package for Social Sciences, version 20.0 (SPSS Inc., Chicago, IL) and the GraphPad Prism Software, version 5.0 (La Jolla, CA, USA). Descriptive statistics were used to provide information regarding the spatiotemporal variables (rainfall, temperature and relative humidity), demographic variables (districts), collection season, collected females and mosquitoes infection by DENV. The Shapiro-Wilk test was used to evaluate whether the data were normally distributed. The asymptotic Pearson’s Chi-square test and the Mann-Whitney *U* test/Kruskal-Wallis test were used to compare percentages and medians, respectively.

Poisson regression models were created to quantify the independent association between the human dengue cases (dependent variable) and the independent variables: grouped into demographic (districts), spatiotemporal [rainfall, temperature (minimum, maximum and average), relative humidity], collection season and field-caught DENV-positive *Ae. albopictus*. Variables with *p*-value < 0.20 in the univariate analysis were selected for multivariate analysis. In each group of variables when more than one variable had *p*-value less than 0.20 hierarchical regression models were created for selection of variables truly associated with the human dengue cases. Variables with p ≤ 0.05 were included in the final model of multivariate linear regression. The Deviance was used to assess the adequacy of the models.

## 3 Results

### 3.1 The field-caught Ae. albopictus relative abundance (RA) by DENV transmission season and by districts

An amount of 511 *Ae. albopictus* mosquitoes were collected in the four-year study period. The field-caught *Ae. albopictus* quantity and percentage across the DENV transmission seasons were extremely variable: 22.8% (n = 117) in the first season, 8.6% (n = 44) in the second season, 39.1% (n = 199) in the third season and 29.5% (n = 151) in the fourth season. There was difference in the proportions of field-caught females among the seasons (p = 0.04): the third had the highest proportion when compared to the second (p < 0.05) (Table 1).

The field-caught *Ae. albopictus* RA varied by district: 234 (45.9%) in Pampulha; 99 (19.3%) in the North; and 178 (34.8%) in the East. There was difference in the proportion of field-caught females among the districts (p = 0.03): Pampulha had the highest proportion when compared to the North (p < 0.05) (Table 1).

### 3.2 The field-caught Ae. albopictus infection rate (IR) by DENV transmission season and by districts

**Fig. 1:**
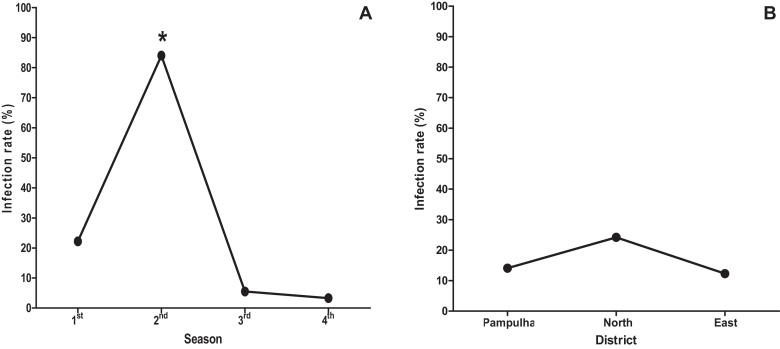
Comparison among the IR of *Aedes albopictus* females (A) by collection seasons and (B) by districts. *p ≤ 0.05.

Across the consecutive DENV transmission seasons the DENV-IR was 15.4% (79 DENV-positive females of the 511 field-caught *Ae. albopictus*). However, there was a significant difference in the proportions of field-caught DENV-infected *Ae. albopictus* when assessed season-by-season (p = 0.02). The second season had the highest proportion of positive females when compared to the fourth season (p < 0.05) (Fig. 1A and Table 2). There was no difference among the proportions of positive females (IR) when comparing the collection districts (p = 0.98) (Fig. 1B and Table 2).

### 3.3 The field-caught Ae. albopictus collected females and climatic variables

When the climatic factors were evaluated, minimum and maximum temperature were correlated with the field-caught *Ae. albopictus* collected females (n = 511) in four different periods and districts, respectively (Table 3). Neither the rainfall nor relative humidity was associated with the field-caught *Ae. albopictus* collected females (Table 3).

### 3.4 The field-caught DENV-infected Ae. albopictus and climatic variables

When the climatic factors were evaluated, none of these variables were correlated with the field-caught DENV-infected *Ae. albopictus* (n = 79) in four different periods and districts, respectively (Table 4).

### 3.5 Multivariate analysis

Variables with *p*-value < 0.20 in the univariate analysis were selected for the multivariate analysis (Table 4). The field-caught DENV-infected *Ae. albopictus*, East district, minimum temperature and relative humidity remained associated with the total number of dengue cases in the multivariate analysis (Table 5).

## 4 Discussion

Currently, *Ae. albopictus* has been detected in different Brazilian regions (Carvalho et al., 2014). As a result of industrial expansion, *Ae. albopictus* has gradually occupied positions in the urban environment of the large cities, as already established by the *Ae. aegypti* species (Pessoa et al., 2013). In the current study, the three districts evaluated – Pampulha, North and East – were situated in an urban area of Belo Horizonte, characterized by high human population density and vast human-built features (houses, commercial buildings, hospitals, schools, paved roads, bridges and municipal parks). In general, *Ae. albopictus* is preferentially found in transitional urban-suburban areas that are closer to parks and woodlands (Rezza, 2012).

In this study, most of the specimens were collected in the third season and in the Pampulha district. The difference in the amounts of *Ae. albopictus* verified among seasons maybe was caused by environmental factors such as space organization and climatic variables as verified by other authors (Lambrechts et al., 2010, Medlock et al., 2012, Carvalho et al., 2014). Regarding the collection district, it is observed that Pampulha has one of the largest green areas of the capital, corresponding to 300,000 m^2^, where is situated the Ecological Park Promotor Francisco Lins do Rego (IBGE, 2014, PBH, 2015). In our investigation, the major quantity of *Ae. albopictus* collected in all of the three districts evaluated, occurred in traps installed near green areas during the four collection seasons (data not described in this article). It is noteworthy that during the fieldwork, independently of the districts evaluated, in the first and in the second seasons, *Ae. albopictus* specimens were detected only in a few traps. Conversely, this situation was completely changed in the third and fourth seasons, when 199 and 151 mosquitoes were captured in the traps, respectively. Based on the findings described above, some authors have considered that *Ae. albopictus* mosquito deserves vigilance. It is crucial to keep in mind that this mosquito can become a bridge between wild and urban cycles of yellow fever and other arboviruses in Brazil, considering its ability to be in wild, rural, suburban and urban environments (Consoli and Lourenço-de-Oliveira, 1994, Lambrechts et al., 2010, Medlock et al., 2012).

In addition, climate is considered the main determinant for the potential distribution of *Ae. albopictus* (Hawley, 1988). In relation to the abundance of field-caught *Ae. albopictus*, in the current study, there was a positive correlation between the numbers of *Ae. albopictus* and temperatures values considering the entire study period. Although there was no significant correlation between the number of *Ae. albopictus* collected and the rainfall, a considerable increase of mosquitoes captured occurred in several moments of this study, especially after the rain peaks (INMET, 2016). In the second season, it was identified the lowest density of *Ae. albopictus* captured. During this season, the rainfall levels were frequently elevated, suggesting that continuous rains could modify the *Ae. albopictus* life cycle (INMET, 2014). The excessive increase of the water level in mosquito breeding sites, in most natural, typical behavior of *Ae. albopictus*, might contribute to the escape of the immature forms from the containers, and consequently the specimens not complete their development. Differing of our findings, Glasser and Gomes (2002) did not observe the rainfall and/or temperature influence on the *Ae. albopictus* dispersion, in a study including field samples of this species in São Paulo State.

In Brazil, the occurrence of human dengue cases is exclusively attributed to the *Ae. aegypti* mosquito and the exact role of *Ae. albopictus* in the DENV transmission in our country, is not already defined. In present study, we selected the three districts which historically had the highest epidemiological impact of dengue epidemics in the city of Belo Horizonte. During the period of the current study, 48,862 (45.6%) of the total of citywide human dengue cases (n = 106,961) were identified in the Pampulha, North and East districts (PBH, 2014). It should be highlighted that a gradual increase in the percentage of total human dengue cases was verified in the third season (84.6%). Concurrently, this season was characterized by the circulation of serotypes DENV-1 and DENV4 in the city (PBH, 2014).

Regarding the second season, the proportion of field-caught mosquitoes achieved the lowest value, especially if this period is compared with the other seasons. On the other hand, the second season was marked by the highest frequency of DENV-infected *Ae. albopictus* females, which achieved the value of 84.0%. Pessanha et al. (2011) valuating three districts of Belo Horizonte (Central-South, East and Venda Nova) verified that 50.0% of larvae of *Ae. albopictus* were infected by *Dengue virus*.

In the epidemic episodes, compared with *Ae. aegypti* (Lambrechts et al., 2010), *Ae. albopictus* is considered less effective as a dengue vector. Conversely, this mosquito was recognized as the main DENV vector in Japan and Taiwan (1945) (Hotta, 1998) and in Macao and Hawaii (2001) (Almeida et al., 2005). Additionally, *Ae. albopictus* has been associated with Chikungunya virus vector in Central Africa (2007) and Italy (2008) (Bonilauri et al., 2008, Pages et al., 2009).

Interestingly, in our study an inverse association between the number of human dengue cases and field-caught DENV-infected *Ae. albopictus* was verified. We speculate that *Ae. albopictus* would be a less efficient DENV vector. The role of this mosquito might be restricted in maintain the *Dengue virus* circulation in this environment. Based in this find, only scarce field Brazilian studies were conducted focusing the role of *Ae. albopitcus* as a DENV vector (Rezza, 2012, Mutsuo et al., 2014). Consequently, further investigations should be developed in order to better understand the relationship between DENV-infected *Ae. albopictus* and human dengue cases.

## Conflict of interest

The authors declare that there is no conflict of interest.

## Figures and Tables

**Table 1:** The field-caught *Aedes albopictus* relative abundance (RA) by seasons and districts.

Collection Season	District N (%)			
	Pampulha*	North	East	All districts
1st	67 (57.3)	22 (18.8)	28 (23.9)	117 (100.0)
2nd	17 (38.7)	13 (29.5)	14 (31.8)	44 (100.0)
3rd*	89 (44.8)	49 (24.6)	61 (30.6)	199 (100.0)
4th	61 (40.5)	15 (9.9)	75 (49.6)	151 (100.0)
Total	234 (45.9)	99 (19.3)	178 (34.8)	511 (100.0)

N (%), number and percentage of field-caught *Ae. albopictus*.

* p ≤ 0.05.

**Table 2:** The field-caught *Aedes albopictus* infection rate (IR) by seasons and districts.

Collection Season	District			
	Pampulha	North	East	All districts
	IF/CF (IR%)	IF/CF (IR%)	IF/CF (IR%)	IF/CF (IR%)
1st	14/67 (20.8)	9/22 (40.9)	3/28 (10.7)	26/117 (22.2)
2nd	15/17 (88.2)	11/13 (84.6)	11/14 (78.5)	37/44 (84.0)
3rd	3/89 (3.3)	4/49 (8.1)	4/61 (6.5)	11/199 (5.5)
4th	1/61 (1.6)	0/15 (0.0)	4/75 (5.3)	5/151 (3.3)
Total	33/234 (14.1)	24/99 (24.2)	22/178 (12.3)	79/511 (15.4)

IF, DENV-infected females; CF, collected females; IR, infection rate (proportion of IF of the CF, multiplied by 100.0%).

**Table 3:** Correlation between field-caught *Aedes albopictus* collected females and climatic variables.

Variables	Climatic variable values	*r*_s_	CI95%	p
CF vs. rainfall	0.10 to 91.40 (mm)	−0.17	−0.34; 0.01	0.06
CF vs. maximum temperature	18.60 to 34.20 (°C)	0.25	0.08; 0.42	0.004
CF vs. minimum temperature	12.10 to 23.20 (°C)	0.34	0.17; 0.49	<0.0001
CF vs. relative humidity	42.75 to 92.75 (%)	−0.13	−0.31; 0.05	0.15

CF, collected females of *Ae. albopictus*; CI, Confidence interval; vs, versus. Source: Climatic variables National, Institute of Meteorology.

**Table 4:** Correlation among field-caught DENV-positive *Aedes albopictus* and human dengue cases and climatic variables.

Variables	Climatic variable values	*r*_s_	CI95%	p
IF vs Rainfall	0.10 to 91.40 (mm)	−0.12	−0.29; 0.07	0.20
IF vs Maximum Temperature	18.60 to 34.20 (°C)	0.08	−0.11; 0.26	0.38
IF vs Minimum Temperature	12.10 to 23,20 (°C)	0.10	−0.084; 0.27	0.27
IF vs Relative humidity	42.75 to 92.75 (%)	−0.12	−0.29; 0.07	0.19

IF, infected females of *Ae. albopictus*; CI, Confidence interval; vs, versus. Source: Climatic variables, National Institute of Meteorology; Human dengue cases, Belo Horizonte Secretary of Health.

**Table 5:** Variables associated with the total number of human dengue cases.

Total of human dengue cases/variables	Univariate analysis		Multivariate analysis				
	PR	p	PR	CI95%-PR	β	CI95%-β	p
DENV-infected *Aedes albopictus* females*	0.36	0.006^a^	0.36	0.17; 0.73	−1.03	−1.75; −0.32	0.005
District							
Pampulha	0.85	0.33	–	–	–	–	–
North	0.54	0.005^a^	0.65	0.41; 1.04	−0.43	0.89; 0.38	0.07
East*	1.65	0.003^a^	1.64	1.18; 2.29	0.50	0.17; 0.83	0.003
Rainfall	1.00	0.92	–	–	–	–	–
Minimum temperature*	0.96	0.005^a^	0.84	0.78; 0.91	−0.17	−0.25; −0.10	<0.0001
Maximum temperature*	0.84	<0.0001^a^	1.01	0.98; 1.05	0.01	−0.25; 0.05	0.54
Average temperature	0.91	<0.0001^a^	1.02	0.95; 1.01	0.02	−0.05; 0.09	0.54
Relative humidity	1.02	0.002^a^	1.02	1.01; 1.04	0.023	0.01; 0.04	0.001

β, Beta; CI, Confidence interval; PR, prevalence ratio; ^a^Variables that entered in the multivariate analysis because showed significance level p ≤ 0.20. The Poisson generalized linear model was adjusted according to the Deviance (P = 0.76).
